# The impact of a mobile geriatric acute team on healthcare consumption

**DOI:** 10.1007/s41999-024-01045-3

**Published:** 2024-09-11

**Authors:** Sofie A. Arvidsson, Karol R. Biegus, Anne W. Ekdahl

**Affiliations:** 1 Department of Geriatric Medicine, Helsingborg Hospital, Charlotte Yhléns gata 10, 251 87 Helsingborg, Sweden; 2 Department of Clinical Sciences, Helsingborg, Faculty of Medicine, Lund University, Lund, Sweden

**Keywords:** Multimorbidity, Acute, Mobile team, Interventions, Healthcare use

## Abstract

**Purpose:**

This study describes the effects of a mobile geriatric acute team (GAT) treating acutely ill geriatric patients in their homes. GAT offered more advanced diagnostic and treatment options than are normally available to primary-care led mobile teams. The aim of this study was to evaluate if interventions by GAT had effect on the number of emergency department (ED) visits, hospitalisations, and length of stay in hospital.

**Methods:**

This is a before–after study, with outcomes recorded for each participant during the 3 months prior to the first visit by GAT and compared to the same outcomes for each participant during the 3 months after the first visit.

**Results:**

The participant’s mean age was 84.6 years, 56% were women. There was no observed difference in ED visits, hospitalisations, and length of stay in hospital for all participants (*n* = 102). However, for the 27 participants living in nursing homes; ED-visits reduced on average by 0.5/participant (*p* = 0.002), the number of hospitalisations reduced by 0.3/participant (*p* = 0.018) and length of stay in hospital reduced by 4.3 days/participant (*p* = 0.045). For the 13 participants referred by ambulance, the number of hospitalisations reduced by 0.7/participant (*p* = 0.044) and length of stay in hospital reduced by 4.1 days/participant (*p* = 0.028). The participants who got intravenous antibiotics also had less hospital care.

**Conclusion:**

This geriatric acute mobile team did not cause reduced hospital care among the participants overall. However, it might have reduced hospitalization in some subgroups, such as patients living in nursing homes or those who got intravenous antibiotics.

## Key summary points

**Aim:**

Does a mobile geriatric acute team (GAT) emanating from a geriatric ward decrease emergency department (ED) visits, hospitalizations and length of stay in hospital?

**Findings:**

There was no observed overall reduction in ED visits, hospitalizations or days spent in hospital for the participants. However, the participants who lived in nursing homes or were referred to GAT by ambulance (in total 39%), and all the participants getting intravenous antibiotics experienced a significant reduction in all these outcomes and consequently a reduction in costs of hospital care.

**Message:**

The results suggest that geriatric acute mobile teams do not reduce ED visits, hospitalisations, and length of stay in hospital overall. However, they might reduce healthcare use in some subgroups.

## Background

Care needs escalate with age, the older population is increasing, and constitutes a growing proportion of hospitalized patients [1–4].

Sweden has the lowest number of available hospital beds per capita in the European Union [5]. In combination with an aging population this has led to overcrowding of wards and emergency departments (ED) in most hospitals. To be in hospital or ED constitutes a risk for the patient’s safety [6, 7] as older patients have an increased risk of confusion, falls, infections, and medication errors [8–11]. People in nursing homes are an especially vulnerable group with a high risk for delirium in new surroundings. There are therefore many reasons to avoid unnecessary hospital admissions for older people.

Sweden’s healthcare system consists of primary and secondary care. Primary care offers first point of contact, work partly as a gatekeeper, and guides the patient through the healthcare system via referrals to specialist care. As in most European countries, a shift has taken place during the latest decades supporting most old people to “age at home”, instead of living in institutions. Home health care, package of care and support from relatives enables continued living at home. Home health care is a range of healthcare services provided in the patient’s home by nurses and assistant nurses employed by the municipality. They, among other things, dress wounds, manage pill organizers, and give subcutaneous injections. Package of care is a package of social services provided by social care staff from the municipality, to support a person in their activities of daily living. In Sweden, the municipalities are responsible for home health care, package of care and nursing home care. Primary care is responsible for physician home visits. It is, however, relatively unusual with home visits by physicians in Sweden and primary care has growing problems with vacancies and care availability. Despite only being about 5% of the population, people over 80 years of age account for 14% of patients seeking hospital care [12]. Especially after the COVID pandemic, several mobile teams have emerged in Sweden in an attempt to prevent the need for hospital care for older people and to compensate for the lack of visits from primary care. This was the case in Helsingborg were an acute mobile team providing geriatric acute medical care was created. This team is called GAT (geriatric acute team) and emanates from the geriatric acute ward at the main hospital. GAT makes home visits for acute medical assessments and treats patients who can and want to be treated at home. Many of these patients have severe frailty and are nearing their end of life. GAT, when referred to, also makes home visits to follow-up patients from the ED or the geriatric ward, to ensure a safe discharge and prevent readmissions.

Both GAT and primary care do home visits to assess patients. There are, some differences between GAT and mobile primary care teams. Primary care physicians make pre-scheduled visits for assessments and meetings to set up a coordinated long-term individual plan for the patient’s further care. GAT makes acute visits. The primary care physicians often make the home visits alone or together with a nurse from home health care. The staff working in GAT always work together, know each other well and rotate between GAT and the acute geriatric ward. GAT has access to more advanced diagnostic and treatment options, such as intravenous treatments, oxygen and bladder scanner. GAT can often make home visits on the day of referral or the day after.

Only a few geriatric mobile teams have been scientifically evaluated, and research in this area is scarce. During 2021 a systematic review of clinical and health economic effects of mobile teams for community-dwelling geriatric patients was done at Örebro University by Camtö [13]. In total fourteen studies were found, of which eight were randomized. Nine had high risk for bias and all but three had high risk for systematic errors. The studies reported contradictory results for the effects of mobile teams on mortality, ED visits, hospitalisations, and length of stay in hospital [13]. Therefore, many questions remain unanswered, and it is important to carry out further evaluations with a scientific approach to bring clarity to the utility of mobile geriatric teams.

The aim of this study is to evaluate if interventions by GAT have effect on the number of ED visits, hospitalisations, and length of stay in hospital.

## Methods

### Trial design

The study is a before–after retrospective study of medical records to access health care utilization and interventions by GAT. The outcomes were recorded for each participant during the 3 months prior to the first visit by GAT and compared to the same outcomes for each participant during the 3 months after the first visit.

### Data collection

The date for the first GAT visit was taken as the inclusion date and all outcomes were calculated from this initial contact. Ethical approval for this study was obtained from The Swedish Ethical Review Authority.

### Study setting

Helsingborg is situated in the south of Sweden and has approximately 150,000 inhabitants [14]. The area contains both rural and urban parts. 9% of the inhabitants were 75 years or older in 2019 [15]. Most health care in Helsingborg and the surrounding municipalities is provided by one hospital and around 25 primary-care centers. Helsingborg Hospital is an acute hospital that admits emergencies 24 h a day. It has approximately 250 available beds and, during on-call hours, a catchment area with 175,000 inhabitants. There is no specialist geriatric outpatient clinic and no private geriatric practitioners in the municipality.

### Participants

Patients that were referred to GAT between 28 April 2022 and 17 October 2022 were considered eligible for the study. Patients that had been in contact with GAT in 2022, prior to 28 April were excluded to allow for a before and after evaluation of GAT’s interventions. To the authors’ knowledge no patient declined to participate, but not all patients were asked to participate. Some staff were not fully informed about the study and therefore did not ask patients to participate.

There was no specific age limit to participate in the study. A few individuals were under 65 years of age, but they all had multimorbidity and were considered in need of an assessment with a geriatric holistic approach. The vast majority were frail older people with multimorbidity.

The 3-month follow-up was considered appropriate since the study participants were older people with unstable medical conditions and short life expectancy.

### Ethical considerations

Before the participants got recruited to the study all received both oral and written information about the study and all left written informed consent. Several participants had cognitive impairments and, in these cases, a relative gave written informed consent.

### Intervention

GAT consisted of a geriatrician or an internal medicine specialist and a nurse and/or a healthcare assistant. GAT assessed and treated geriatric patients in their home environment and aimed to take a holistic view of the patient’s situation. For instance, GAT contacted social-care managers for initiating or increasing package of care and contacted primary care for initiating home health care services when needed. If required, GAT could refer the patient to a pharmacist, physiotherapist, and occupational therapist. GAT had a broad collaboration with home health care, the ambulance service, primary care, social care managers and worked as a bridge between the primary and secondary care.

GAT worked only office hours. Referrals were received from health care providers in home health care, nursing homes, the ambulance, the ED, geriatric ward, and primary care. As far as possible most GAT visits were done the same day as the referral. The ambulance services or ED could refer patients out of hours by booking them into GATs available time slots for the next day, which guaranteed that patients will be seen by GAT the next day. Most referrals concerned patients in need of an acute medical assessment or intervention to avoid an ED visit or hospitalization. If patients under GAT’s care needed attention out of hours, they could call the ambulance or a single responder doctor service, this was available to all inhabitants in the area.

GAT provided medical assessments, blood tests, and contact with relatives and nurse/social care staff. A bladder scanner and arterial doppler were available when needed. GAT had the ability to take all blood tests or cultures that were possible to take at the hospital; and the results of blood tests were available within an hour. Available treatment options included oxygen administration through an oxygen concentrator, blood transfusions and other intravenous (i.v.) treatments (such as fluids, antibiotics, diuretics, iron, osteoporosis medicines and blood). GAT always did a medication review. The duration of GAT’s interventions and number of follow ups varied from one to several visits depending on the patient’s needs, but the time for follow-up was often not more than a couple of weeks. After the acute issue and when the situation was stabilized, the patient was referred back to primary care.

### Outcomes

The registered outcomes were the number of ED visits, number of hospitalizations, length of stay in hospital, initiation of home health care or package of care, increasing home health care or package of care, number of visits by doctor, telephone follow-ups by doctor, i.v. fluid therapy and other i.v. treatments (such as antibiotics, diuretics, i.v.-administered iron, or blood transfusions). It was also specified whether the patients were community-dwelling or were living in a nursing home and which care actor referred the patient to GAT.

We registered mortality in the 3-month follow-up period.

### Statistical analyses

The results are presented as descriptive statistics and a before- and after analysis with paired sample *T* test. The results are presented as statistically significant if *p* < 0.05.

## Results

### Participants and baseline characteristics

102 patients were included in the study. The mean age among the participants was 84.6 (51–100) years. Of all participants 56% were women, 73% lived at home and 27% lived in nursing homes. 78% of those living at home had help from a package of care or a social alarm (an alarm you wear, to push when in need of help e.g., after a fall. The alarm is connected to a department at the home services that responds 24/7). Half of the participants living at home (51%) had home health care. During follow-up 26% deceased. Three of the participants were admitted directly to the geriatric ward.

GAT gave i.v. treatments (i.v. fluids, antibiotics and infusions) 84 times to 38 participants, and gave 28 blood transfusions to 16 participants. About half of the participants (52%) of those living in nursing homes got i.v. treatment, compared to 34% of the participants living at home. In 37% of cases GAT increased the combination of home health care and package of care. For details and interventions in subgroups see Table 1.

**Table 1:** Interventions made by the geriatric acute team

Participants (*n*)	Visits per patient (mean)	I.V. fluids (%)	I.V. antibiotics (%)	I.V. iron/diuretics (%)	I.V. blood (%)	GAT started HHC (%)	GAT increased HHC/POC (%)
All participants (102)	3.0	25	26	32	27	21	37
Lived at home (75)	3.4	17	20	35	17	29	51
Lived in nursing home (27)	2.3	44	41	37	56	0	0
Referred by primary care (38)	4.8	37	48	78	52	41	56
Referred by ambulance staff (13)	3.1	38	46	0	8	38	62
Referred by nursing home staff (15)	2.1	27	13	47	47	0	0
Referred by HHC nurse (22)	2.9	14	23	23	18	0	36
Referred from the ED (9)	1.9	0	0	22	0	44	33
Referred from GAW or another ward (5)	6.0	60	0	20	50	20	40
Deceased during follow-up (26)	3.3	31	50	69	31	35	35

*I.V* intravenous, *GAT* geriatric acute team, *HHC* home health care, *POC* package of care, *ED* emergency department, *GAW* geriatric acute ward

### Outcomes

There was no difference in ED visits, hospitalisations, and length of stay in hospital for all participants, but these outcomes all decreased for patients living in nursing homes and for participants when GAT was initiated by the ambulance (in total 39% of the participants). There was also a decrease in all outcomes for the participants who got i.v. antibiotics and in ED visits for the participants who got i.v. fluids. See Tables 2, 3 and 4.

**Table 2:** Mean number of visits to the emergency department 3 months before and 3 months after the first visit by the geriatric acute team

Participants (*n*)	No of ED-visits before Mean (SD)	No of ED visits after Mean (SD)	*p* value
All participants (102)	0.7 (0.9)	0.5 (0.8)	0.323
Living in nursing homes (27)	0.6 (0.8)	0.07 (0.3)	0.002*
Living in nursing homes (deceased excluded) (20)	0.6 (0.8)	0 (0.0)	0.004*
Living at home (75)	0.7 (0.9)	0.7 (0.7)	0.78
Living at home (deceased excluded) (56)	0.7 (1.0)	0.6 (0.9)	0.59
Referred by ambulance staff (13) (living at home)	1.1 (0.8)	0.5 (1,0)	0.131
GAT-intervention with iv antibiotics (11)	1.0 (0.9)	0.09 (0.3)	0.01*
GAT-intervention with iv fluid (14)	0.9 (0.8)	0.07 (0.3)	0.001*
GAT-intervention with blood transfusion (16)	0.7 (0.7)	0.5 (0.8)	0.509
GAT initiating HHC(17)	0.8 (1.3)	0.7 (1.0)	0.778
GAT increasing HHC or POC(34)	0.7 (0.7)	0.9 (1.0)	0.362
Deceased (26)	0.7 (0.7)	0.8 (1.0)	0.557

*ED* emergency department, *SD* standard deviation, *GAT* geriatric acute team, *HHC* home health care, *POC* package of care

*p ≤ 0.05

**Table 3:** Mean number of hospitalisations 3 months before and 3 months after the first visit by the geriatric acute team

Participants (*n*)	Number of hospitalisations before, mean (SD)	Number of hospitalisations after, mean (SD)	*p* value
All participants (102)	0.4 (0.7)	0.4 (0.7)	0.621
Living in nursing homes (27)	0.4 (0.6)	0.07 (0.3)	0.018*
Living in nursing homes, deceased excluded (20)	0.3 (0.6)	0 (0.0)	0.030*
Living at home (75)	0.4 (0.8)	0.5 (0,7)	0.323
Living at home, deceased excluded (56)	0.5 (0.7)	0.3 (0.6)	0.36
Referred by ambulance staff, living at home (13)	1.0 (0.9)	0.3 (0.5)	0.044*
GAT-intervention with iv antibiotics (11)	0.7 (0.6)	0.09 (0.3)	0.011*
GAT-intervention with iv fluid (14)	0.6 (0.6)	0.1 (0.4)	0.054
GAT-intervention with blood transfusion (16)	0.4 (0.6)	0.4 (0.6)	1.00
Deceased (26)	0.5 (0.6)	0.8 (0.8)	0.175

*SD* standard deviation, *GAT* geriatric acute team, *iv* intravenous

*p ≤ 0.05

**Table 4:** Mean number of days in hospital 3 months before and 3 months after the first visit by the geriatric acute team

Participants (*n*)	Days in hospital before, mean (SD)	Days in hospital after, mean (SD)	*p* value
All participants (102)	3.4 (7.0)	3.5(7.1)	0.919
Living in nursing homes (27)	5.1 (11.0)	0.8 (3.0)	0.045*
Living in nursing homes, deceased excluded (20)	3.4 (7.7)	0 (0.0)	0.064
Living at home (75)	2.7 (4.6)	4.4 (7.8)	0.11
Living at home, deceased excluded (56)	2.6 (4.6)	3.3 (7.5)	0.55
Referred by ambulance staff, living at home (13)	6.2 (6.0)	2.1 (3.6)	0.028*
GAT-intervention with iv antibiotics (11)	5.9 (7.7)	0.4 (1.2)	0.045*
GAT-intervention with iv fluid (14)	4.7 (5.8)	1.1 (2.9)	0.062
GAT-intervention with blood transfusion (16)	5.4 (12.1)	2.5 (4.1)	0.323
Deceased (26)	4.8 (9.9)	6.3 (7.6)	0.553

*SD* standard deviation, *GAT* geriatric acute team, *iv* intravenous

*p ≤ 0.05

There was an overall mortality of 26 participants (26%) and it was the same percentage whether participants were living at home or in nursing homes.

## Discussion

The present study adds to the knowledge of effects of mobile teams. The main finding was that the difference in health care utilization when GAT took care of patients in need of more advanced interventions such as i.v. antibiotics and i.v. fluids, as we often did in nursing homes, or when called upon by ambulance. Normally primary care does not give i.v antibiotics in nursing homes, and do not answer to acute calls from the ambulance. The geriatric mobile team was also a part of a geriatric hospital ward which meant that the staff in the team also worked in the geriatric ward. This strengthened their competence and skills to give more advanced treatments and investigations and is probably part of the explanation for the results. Also, this increases the teams’ knowledge of what can be offered in hospital, when the patients would benefit from hospitalization and why some conditions could be treated just as well at home despite the patient having poor vital signs or laboratory findings.

There was no significant reduction in ED visits, hospitalisations or length of stay in hospital for all participants seen over 3 months after GAT’s first visit. One explanation for this outcome might be that, if we assume the patients were frail with a high risk of disease exacerbations and functional decline, this could explain the occurrence of secondary illnesses and ED visits, hospitalisations and longer hospital stays [16]. The frailty of the participants is suggested by the high proportion that lived in nursing homes, had home health care and/or package of care. Second, the geriatric mobile team might have discovered previously unidentified diseases and unmet needs which led to higher care utilization. Third, some interventions done by the team were not aimed at hospital avoidance, but rather routine care offered at home simply because the patient could not be seen by primary care such as for example blood transfusions. Finally, the team physician was either a geriatrician or an internal medicine specialist. It might be the case that all team members need specific geriatric training for the team to effectively reduce hospital care.

When we combined the outcomes for the participants living in nursing homes with the patients referred from ambulance there was no overlap in participants and there was an even more pronounced effect. The results remained significant when we excluded the participants that deceased during the observation period. These groups received primarily interventions aimed at hospital avoidance and they were often on the brim of needing hospital care. The results suggest that in these cases it is beneficial to use a mobile team with the experience of more advanced interventions and with direct access to the hospital or the geriatric ward in order to avoid hospital care.

From a health economic perspective, the decrease of length of stay in hospital for some of the participants is an important finding. In Sweden the daily cost for a hospital bed lies around 915€ per day. In this study we saw a mean decrease in 4.1 days per participant of the participants living in nursing homes or referred by ambulance which corresponds to around 3751€/participant over the 3 months the study was ongoing. The costs of GAT/year was roughly 600,000€ for around 2100 visits or 285€/visit by the team. The patients in nursing homes and referred by ambulance had in total 102 visits for the 40 participants (Table 1) to an estimated cost of 727€/participant. The decrease in hospitalization resulted in savings of 3751–727 = 3024€/participant living in nursing homes or referred by ambulance. This is a total of around 120,000 € saved for these groups during the 3 months follow-up.

In addition, GAT reduces the load on overcrowded EDs and hospital wards and thus improves the working environment—an important “side-effect” of advanced mobile teams together with the fact that most patients want to avoid hospital visits [17].

GAT made an average of 2.95 visits per patient. In a Swiss study similar results were found, where patients connected to a geriatric mobile team had more consultations but fewer of these led to hospitalisations in comparison to patients receiving ordinary care [18]. The results of our study could be compared to other studies on mobile teams. One earlier Swedish randomized controlled study (The Age-FIT-trial) showed a higher sense of security and fewer days in hospital when care was given by a geriatric team versus usual care [19]. Yet another Swedish study with a geriatric mobile team resulted in no difference in hospital care and an increased number of contacts with physicians [20]. In future it might be important to differ mobile teams according to their ability of more advanced intervention when the goal is to decrease health care consumption, but when the goal is good continuity and planned care it is probably better to have mobile teams based in primary care.

As we are dealing with frail older people it is expected for them to get worse and need more health care—or die—over time. The results of our paper showed the opposite result which implies that GAT really made a difference for some groups of participants. Since this study takes place in a real-life setting, it is likely for the result to be generalized to other mobile teams who work in similar ways and in a similar setting as GAT.

One limitation of this study is that not all patients assessed by GAT during the study period participated in the study. Some were not asked to participate because the team forgot to ask them. High workload and change of physicians were not uncommon, where some physicians had no or little knowledge of the study. In this sense, it is not likely that there has been a conscious selection of patients. To the authors’ knowledge, no patient that was asked declined to participate. Conversely, the hardest situations to include patients in, was probably acute and emotional situations, i.e., when there was acute need for deciding about palliative care at home or further curative treatment at the hospital. GAT has probably been less prone to ask about study participation in these cases. This might affect the results on the number of deaths, ED visits and hospitalisations.

For the results to be more generalized a larger, preferably multicentre, study is needed. The sub-group analysis was not pre-specified and had a small number of participants. These findings need to be confirmed by a study in these specific populations. It is also important to distinguish between mobile teams emanating from hospital or primary care and further study which group of participants have the greatest benefits of more advanced care at home.

## Conclusion

The geriatric acute team did not cause reduced hospital care among the participants. However, the results suggest that with access to more advanced interventions than primary care and a shorter response time it might reduce ED visits, hospitalisations, and length of stay in hospital for patients living in nursing homes and patients referred by ambulance and even seemed to provide a health economic benefit in these groups. Further research is needed to confirm the results.

## Data Availability

The datasets generated and analyzed during the current study are stored by Anne Ekdahl and available from the corresponding author on reasonable request.
